# Supplementary material for: Dissecting heritability, environmental risk, and air pollution causal effects using > 50 million individuals in MarketScan
Source: Nat Commun. 2024 Jun 25;15:5357. doi: 10.1038/s41467-024-49566-6 (PMC11199552; doi:10.1038/s41467-024-49566-6)
Supplement: Supplementary file 1 — Supplementary Information [file 41467_2024_49566_MOESM1_ESM.pdf]

Supplementary Figures

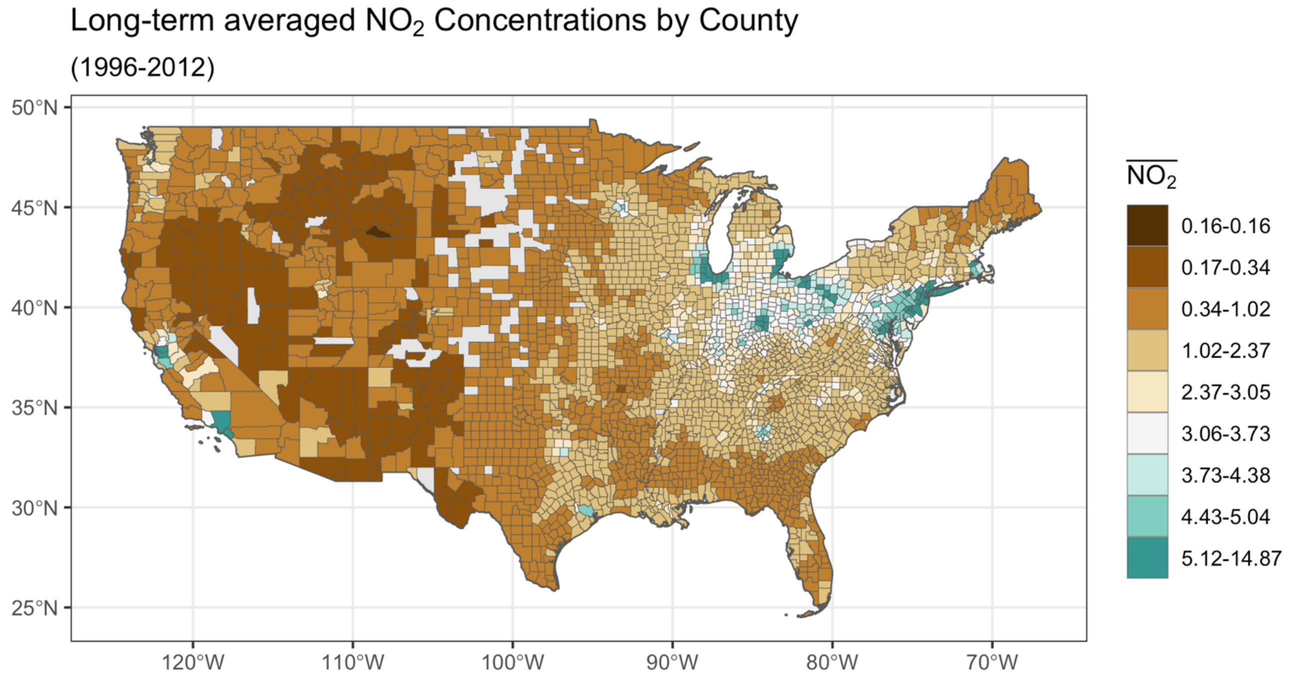

Supplementary Figure 1: **Long-term averaged NO_2 Air Pollution by county.** The average concentrations of NO_2 by county are displayed, where pollution for each year was extracted at the latitude and longitude of the centroid of each county from satellite-based estimates of NO_2 [1, 2].

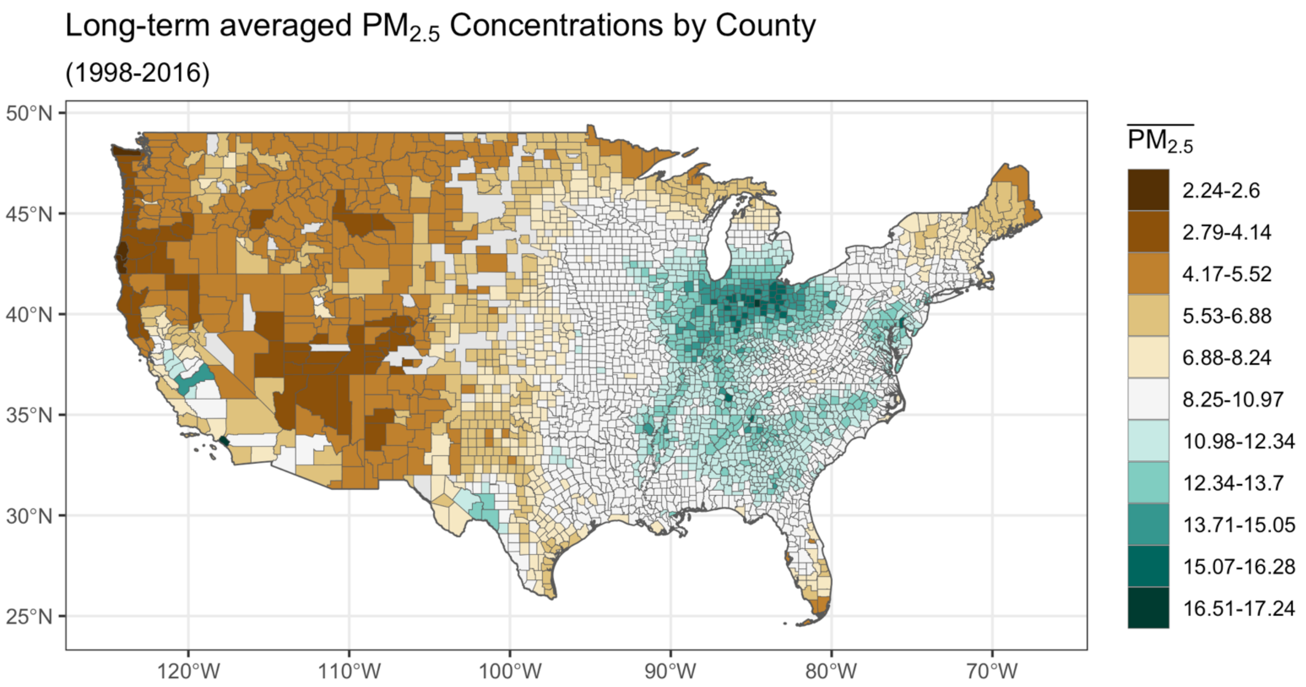

Supplementary Figure 2: **Long-term averaged $PM_{2.5}$ Air Pollution by county.** The average concentrations of $PM_{2.5}$ by county from 1998-2016 are shown, where pollution for each year was extracted at the latitude and longitude of the centroid of each county from satellite-based estimates of $PM_{2.5}$ [3, 4].

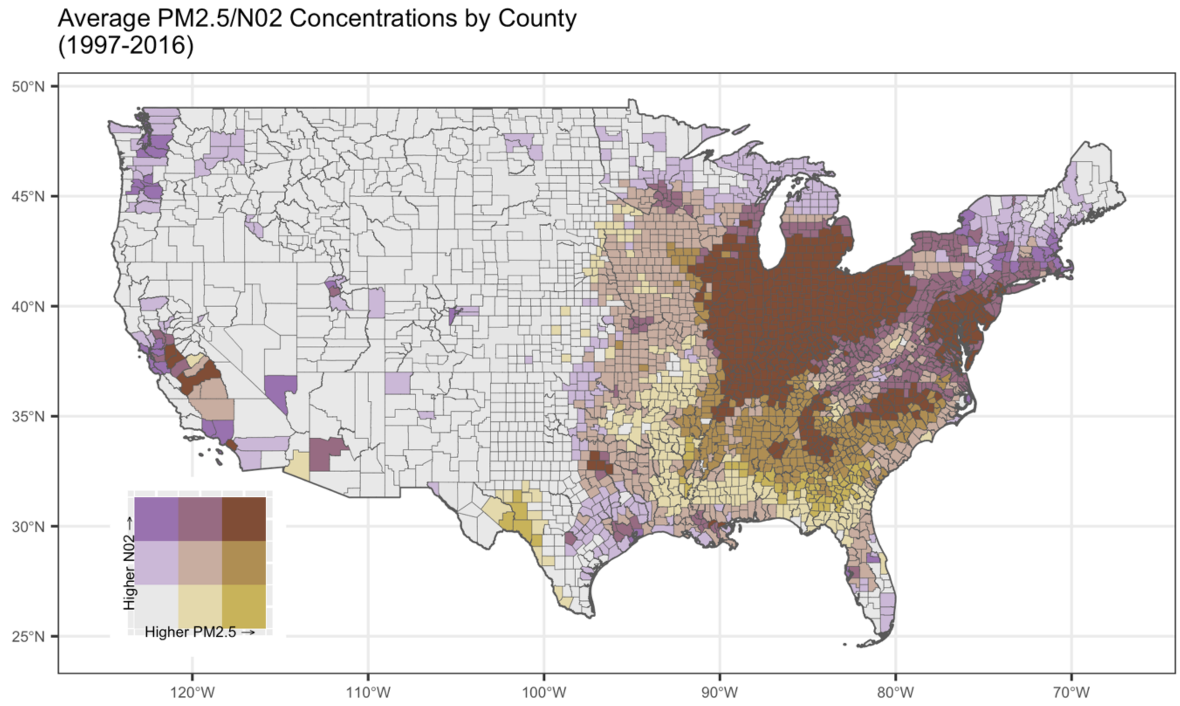

Supplementary Figure 3: **Bivariate distribution of long-term averaged NO_2 and $PM_{2.5}$ air pollution.** We stratified the long-term average of NO_2 and $PM_{2.5}$ air pollution values into 3 equal-sized bins, using cutoffs at 33th and 67th percentiles for each pollutant (i.e., $NO_2 < 0.9$, $0.9 < NO_2 < 1.76$, and $NO_2 > 1.76$; $PM_{2.5} < 8.63$, $8.63 < PM_{2.5} < 11.1$, and $PM_{2.5} > 11.1$). Each county/MSA was assigned a color based on the bin that their NO_2 and $PM_{2.5}$ levels belong to. Individual maps for NO_2 and $PM_{2.5}$ are provided in Supplementary Figures 2-3.

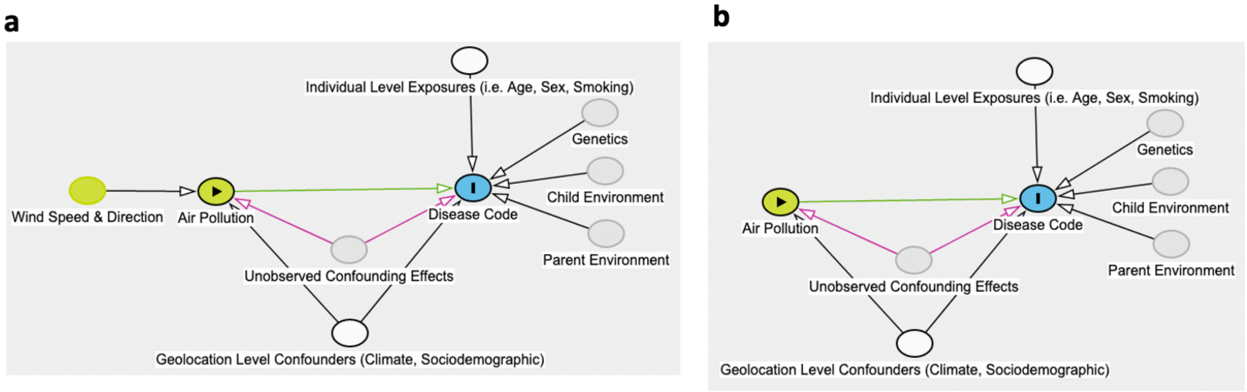

Supplementary Figure 4: Directed acyclic graph for estimating causal effect of pollution on disease. In **panel a**, we describe the conceptual SMILE-2 model that uses wind-direction as an instrument for estimating the causal effects of NO_2 and $PM_{2.5}$ air pollution on diseases. Wind speed and direction satisfy the relevance and exogeneity condition for instrument variables and are unlikely to cause any disease directly. It hence provides more robust estimates of the causal effect in the presence of confounders. **Panel b** describes a standard regression model for association analysis which may be subject to the impact of confounders**.** The direct causal effect of air pollution cannot be properly assessed in the presence of unobserved confounding effects without the use of instrumental variables.

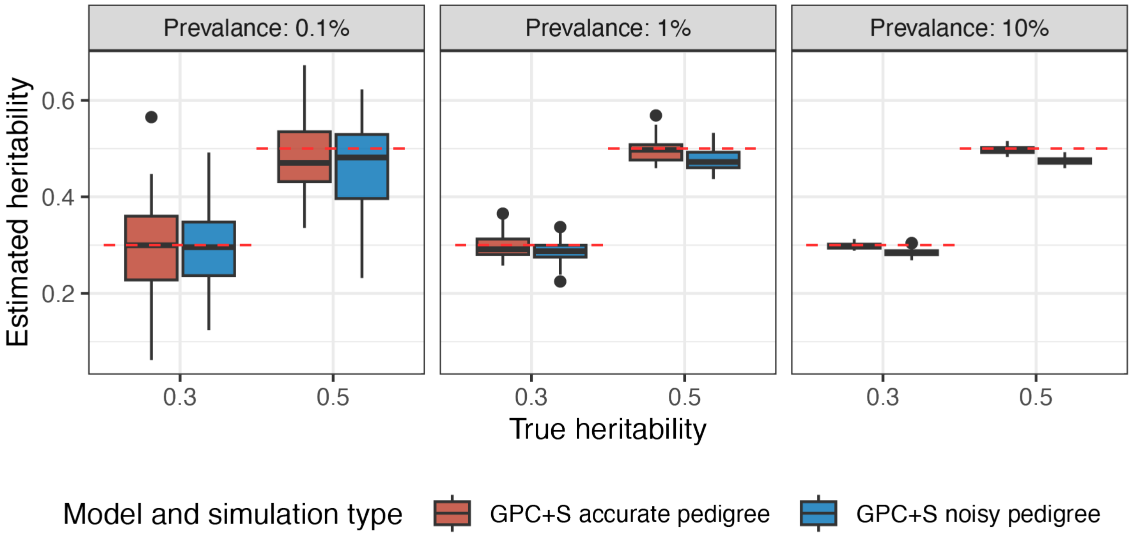

Supplementary Figure 5: Simulation evaluation of heritability estimation in the presence of mis-specified pedigrees. Among simulated families of each replicate, 2.4% have 1 or 2 adoptive children and 6.2% have 1 or 2 stepchildren, which reflects estimates from US census. We fit SMILE (GPC+S) model using the true genetic relationship matrix and using the mis-specified genetic relationship matrix, where all children were treated as biological children of both parents. We consider scenarios with different combinations of genetic heritability (G), and parental (P), children (C) and spatial community-level (S) environment, as well as different phenotype prevalence. A total of 250,000 nuclear families were simulated under each scenario with 20 replicates. The heritability estimates using “accurate pedigree” and “noisy pedigree” are very comparable across all scenarios. Each dot represents the estimated genetic heritability across simulation replicates under different scenarios. Minima and maxima values (excluding outliers) are represented by the lower- and upper-bound of the whiskers. Median value is represented by the bold line in the middle. First and third quartiles are represented by the lower- and upper-bound of the box.

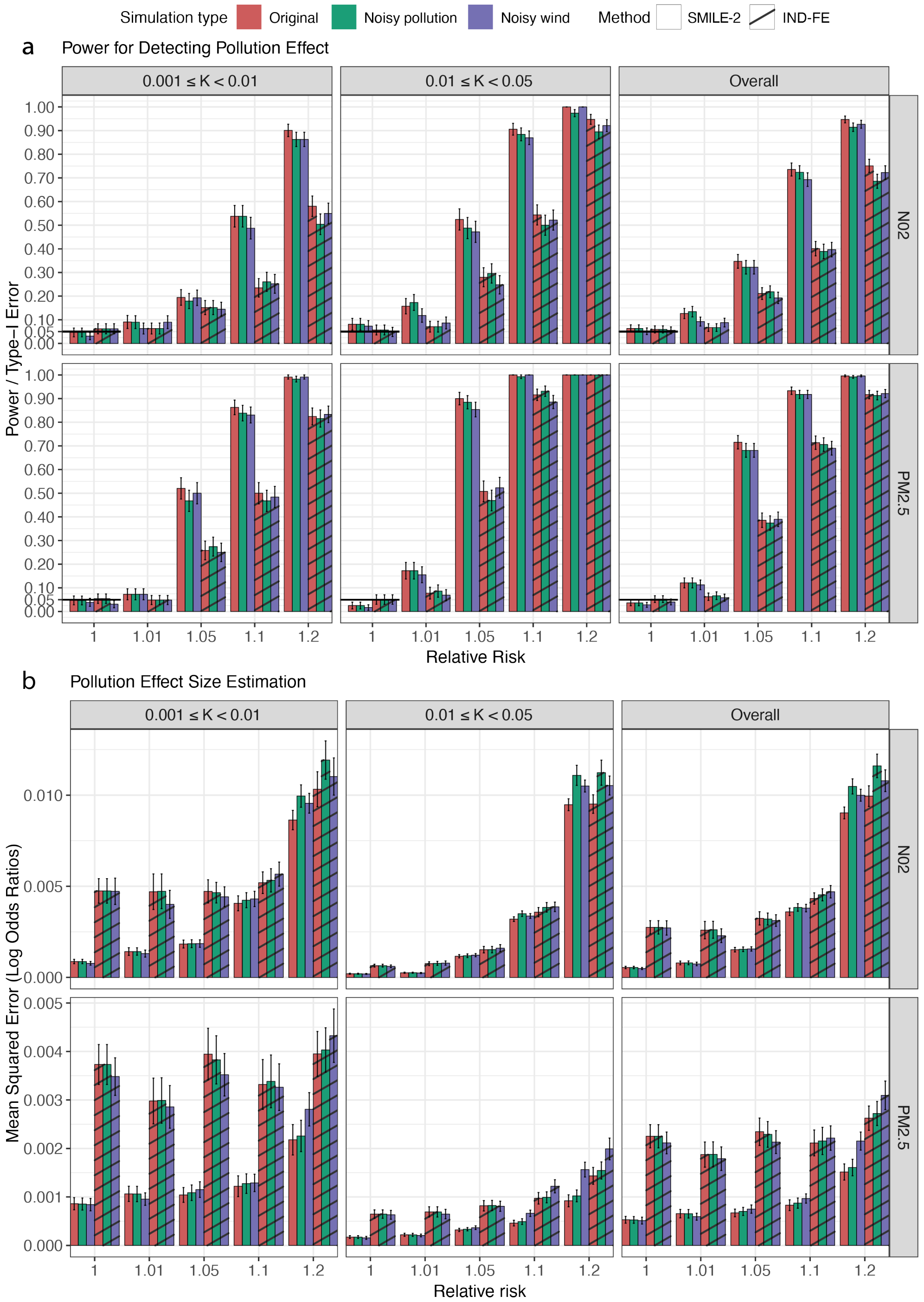

Supplementary Figure 6: Simulation evaluation of causal effect estimates in SMILE-2 using noisy pollution and wind measurements. For each location, we create noisy wind and pollution measurements by randomly sampling measurements from 10 nearest neighboring locations in place of the actual measurement. We assessed how the noisy measurements impacted the type-1 error, power, and mean squared error (MSE) of the estimated log-odds ratios. In our simulation, we resampled with replacement families from MarketScan as well as the confounder variables associated with those families. We varied the causal effect (in the unit of relative risk) for $PM_{2.5}$ and NO_2 between 1.0, 1.01, 1.05, 1.1, or 1.2. Confounding effects, genetic and family environment variance components were simulated based on the parameter estimates from the analysis of MarketScan data. The binary disease status was obtained by dichotomizing the continuous liability threshold according to the disease prevalence from real data. The type I errors, power (panel a) and mean squared errors (MSE) of the causal effect estimates (panel b) were evaluated using 10 replicates for each of 200 randomly selected PheWAS codes. We compared the causal effect estimates using original and noisy wind and pollution measurements. Results stratified by disease prevalence (K) and pooled results are shown. The error bars represent the standard error (SE) across simulation replicates performed under different scenarios.

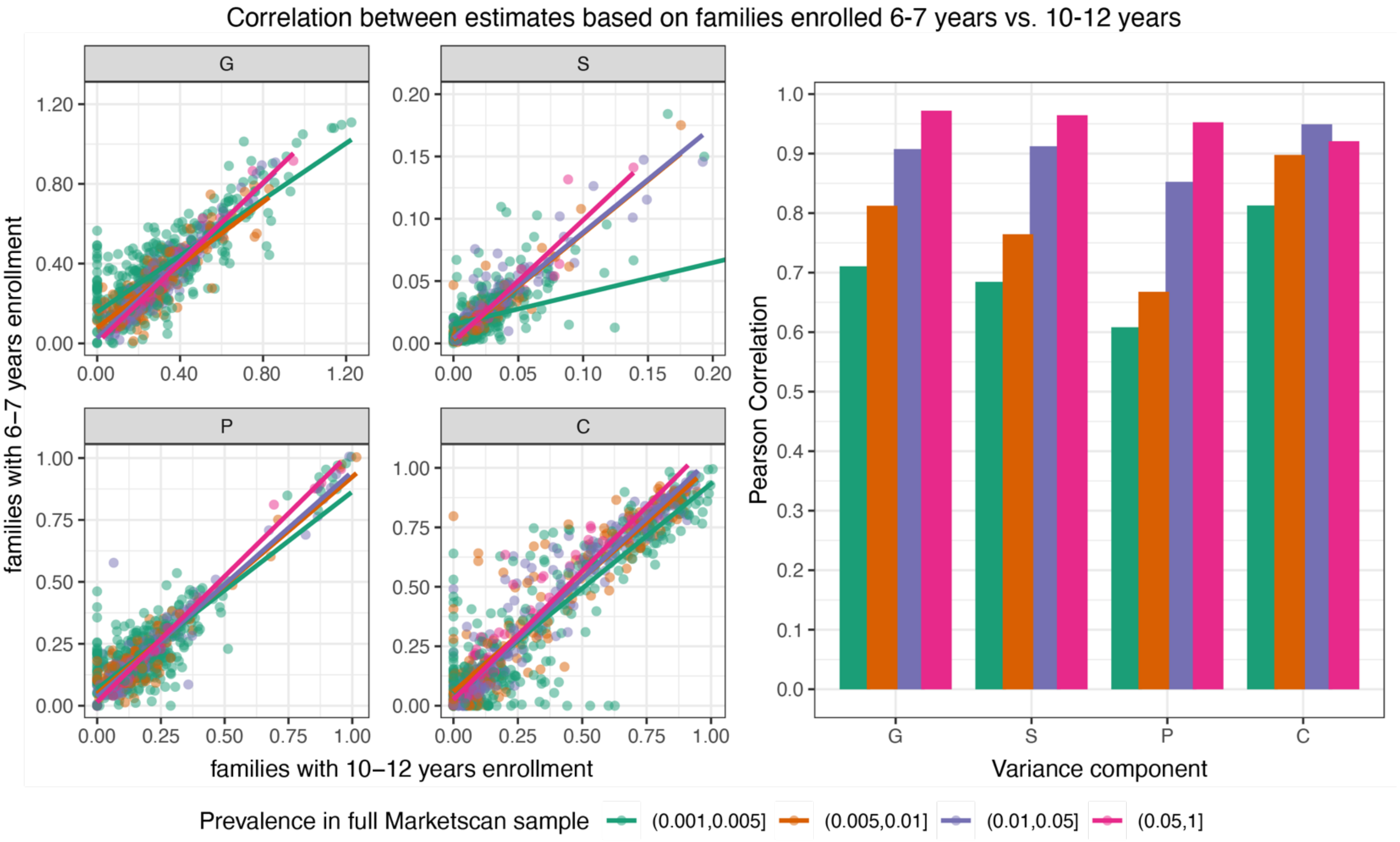

Supplementary Figure 7: Correlation in SMILE Variance Component estimates comparing families based on length of enrollment observed in MarketScan dataset. We refit SMILE models using families enrolled in the MarketScan database for only 6-7 years, and for families enrolled for 10-12 years and compare the genetic (G), parental (P), children (C) and spatial community-level (S) environment variance components estimates. Correlation was high for each of the variance components, particularly for diseases with prevalence > 0.01. The correlation is only slightly lower for diseases with prevalence ≤ 0.005, as the estimation can be unstable when the number of disease cases in the dataset becomes small.

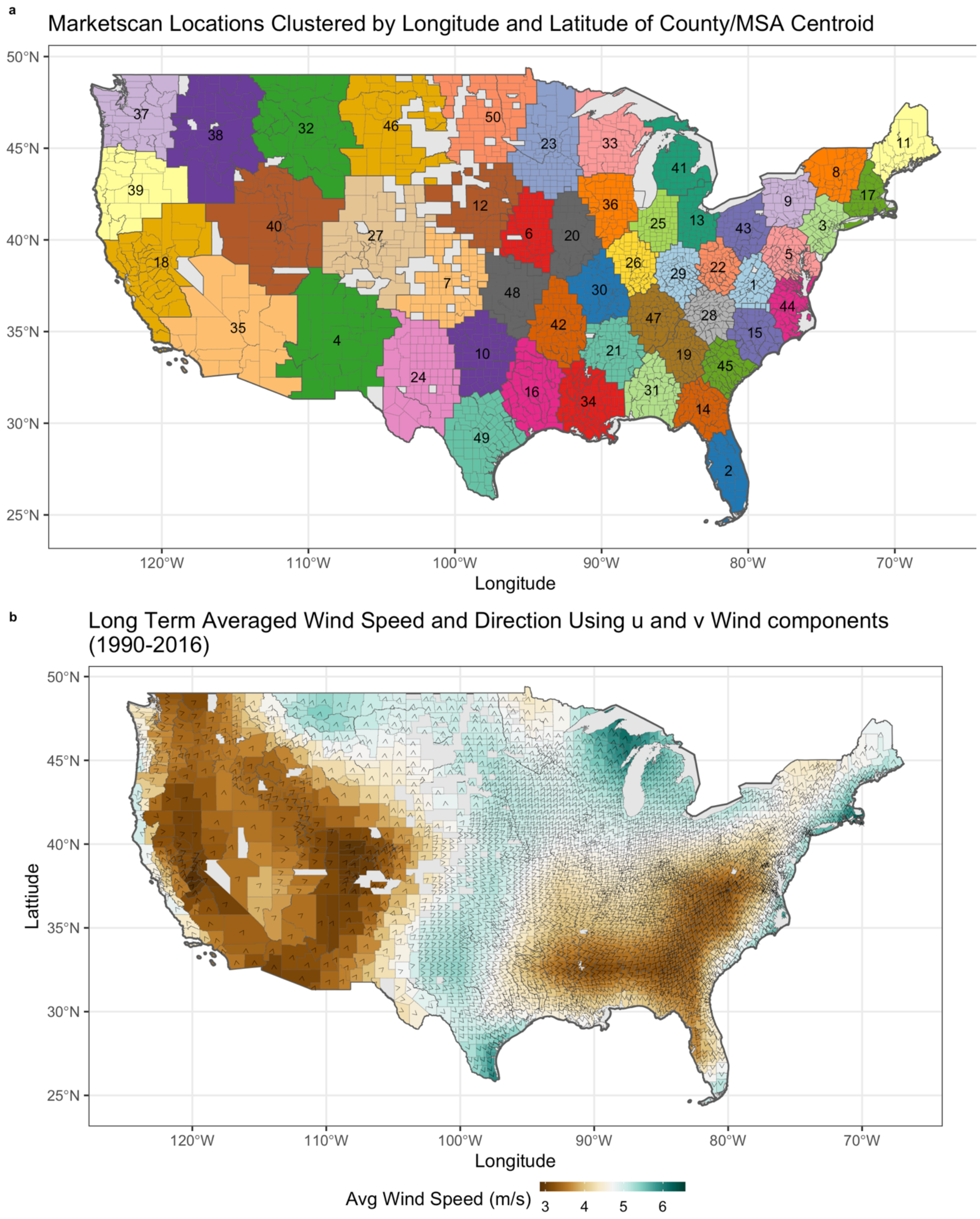

Supplementary Figure 8: Construction of instrument variables for assessing the causal effect of air pollution using geographic clusters, local averaged wind speed and directions. Stage 1 regression model creates the instrument variable for pollution via the interaction between local geographic cluster membership. Cluster information is shown in (**panel a**), with the numbers on the map indicating the cluster membership. The long term averaged wind speed (as represented by the color) and direction (as represented by the direction of the wedges) at each MarketScan location are shown in (**panel b**).

Supplementary Methods

1. **Description of datasets**

MarketScan Dataset
We used the inpatient and outpatient records from the IBM MarketScan health insurance claims database from 2005 to 2017. Besides their health records, patient records include individual level sex, year of birth, a geolocation within the United States, and the relationship to the employee who is the primary enrollee of the insurance policy (i.e., Employee, Spouse, or Child/Other).

Disease Outcomes
Cases and controls for disease outcomes were defined according to the PheWAS code [5]. PheWAS codes are groupings of ICD9/10 insurance billing codes constructed to represent biologically/medically relevant phenotypes. For a given PheWAS code phenotype, we identified as cases any individuals who had at least one of the corresponding ICD9/10 codes during their recorded billing history.

Approximate Familial Relationships
We inferred family structure from the relationships of each enrollee to the designated policy holders in each family [6], and performed stringent data quality checks to ensure the accuracy of the inferred familial relationships. Specifically, we assume that "Employee" and "Spouse" are the biological parents of the enrollees encoded as "Child/Other" within the family. We selected for analysis nuclear families which were enrolled for at least 6 years within the database. We also required that the youngest sibling in each selected family be at least 10 years old at the beginning of their 6+ year enrollment period. To maximize the validity of the assumed biological relationships within a family, we removed families where the "Employee" and "Spouse" were of the same sex, and families where either "Employee" or "Spouse" were not older than the "Children" by at least 14 years. We also removed families where the designated "Employee" had multiple "Spouses" with different enrollment ID's, as in this situation it is unclear which Spouse (if any) may be the biological parent of the children. This curated dataset consisted of 257,620 families for which we carried forward the downstream analyses.

Geographic Locations in MarketScan
To model community-level environment contributions, we made use of geolocations embedded in MarketScan. Prior to 2014, the geolocation of each individual was given as a county code, while during 2015-2017, the geolocation was provided by a code indicating Metropolitan Statistical Area (MSA). Only individuals with county or MSA level geospatial information were retained for analysis. A single geolocation was chosen to represent each individual. As county code locations are more specific than MSAs (which typically encompass multiple counties), the most frequent county code location was used for each individual wherever possible as the representative geolocation. For individuals where only MSA was provided, the most frequent MSA was chosen as their geolocation for further analyses. We included geolocations within the 48 continental U.S. states and Washington D.C., but excluded Alaska, Hawaii, and non-state U.S. territories due to the complexity of modeling their spatial dependence with continental states.

Environmental Exposure Information from External Datasets
We integrated county and MSA-level environmental and sociodemographic exposures using public datasets to assess the causal effects of air pollution on disease. We focus on pollution levels that are broadly measured across the country. Pollution exposures include particulate matter 2.5 ($PM_{2.5}$) [3, 4] and nitrogen dioxide (NO_2) [1, 2]. We also used the median income, population density, maximum education level, and race distribution variables from the 5-year ACS Community Survey 2015 [7] as covariates to control for confounding in regression models when assessing pollution effects. Wind speed and direction [8] were used as instrumental variables for causal inference. We summarized all covariates at the county and MSA location level in Supplementary Data 7. The fields extracted from the 5-year ACS Community Survey 2015 are listed in Supplementary Data 8.

2. **Simulations**

Variance Component Model Simulation

We simulated binary phenotypes under a liability threshold model where explainable variation was attributed to varying proportions of parental-environment, children-environment, additive genetic, and spatial variance. Specifically, we simulated 5 replications of each combination of the following liability scale variance component values: $\sigma_s^2 \in \{0, 0.01, 0.05\}$, $\sigma_g^2 \in \{0, 0.3, 0.5\}$, $\sigma_{par}^2 \in \{0, 0.05, 0.1, 0.2\}$, and $\sigma_{child}^2 \in \{0, 0.05, 0.1, 0.2, 0.6\}$, which reflect the distribution of estimated variance components in the MarketScan data (Supplementary Data 2). CAR covariance was used for all scenarios with spatial random effects, as it is the most frequently chosen spatial covariance structure in MarketScan data analysis, and we found that estimated heritability is robust to the mis-specified spatial covariance structure (Figure 3C). For each simulation setting, we considered sample sizes of 50,000 and 250,000 nuclear families. Family locations were sampled with replacement from real MarketScan locations, so that the fraction of families from each location in the simulated data reflects the real distributions. After generating the underlying standard-normally distributed liabilities, we created binary phenotypes under the liability threshold model for population prevalence's of 0.1%, 0.5%, 1%, 5%, 10%, and 40%. We then fit eight linear mixed models to each scenario with different combinations of random effects, including *GPC+S (SMILE), GP+S, GC+S, GPC, PC+S, PC, G+S,* and *S* models. For each simulated dataset, we fitted different sub-models using Laplace approximation, and evaluated the bias and the mean squared error for each parameter estimate using different sub-models. To evaluate the effectiveness of BIC for selecting the best model, we identified the proportion of replicates where the correctly specified sub-model had the lowest BIC (Figure 1A). The full simulation results are summarized in Supplementary Data 1.

Pollution Causal Effect Models Simulation
We ran extensive simulations to evaluate the properties of our pollutant effect estimates. To generate data under realistic scenarios of confounding, we resampled families from MarketScan together with all individual specific covariates, and simulated phenotypes using the estimated model parameters from our real-data analysis.

Specifically, we generated pseudo-phenotypes for all of our 1,083 traits. We randomly chose half of the traits and assume that the pollutants have no causal effect on them. We assume that air pollution causally influences the other half of the traits. The relative risks of air pollution for each trait (as defined by the SMILE-2 model equation (S12)) were randomly sampled from 1.01, 1.05, 1.1, or 1.2 with equal probability. The half without causal effects were used to evaluate type I error and the other half with causal effects were used to assess power. The confounder effects (e.g., social economic status, average temperature, etc.) were simulated based on estimates from the MarketScan data analysis. As all individuals were sampled from real locations, they are subject to the influence of location-specific confounders such as social economic status and climate, which may be correlated with pollution levels. For each PheWAS disease code, we approximated the relative risks of confounders on the liability scale using the fixed effects estimated from the MarketScan data and the detailed formulae are derived in Section 8 of the Supplementary material. We repeated this process six times for each of the 1,083 MarketScan phenotypes and made sure that each phenotype was chosen in the simulation for both the null (RR=1) and alternative (RR > 1) hypotheses at least once.

For each PheWAS code-based phenotype, we first sampled 250,000 nuclear families with replacement from our MarketScan family cohort, retaining both individual-level and location-level covariate information. After the underlying liability was generated, the binary disease status was created by dichotomizing the liability according to the threshold set to match the observed prevalence in the MarketScan data. We fit the SMILE-2 model (as in equation (S12)) to the simulated datasets and estimated the type-I error, power, and the MSE of the estimated log-odds ratios for different phenotypes and pollution-effects sizes.

We compared our SMILE-2 model to an 'independent subject' fixed effect model (IND-FE), which uses unrelated individuals as input and does not account for genetics or family-level environment.

3. <u>Comparison of SMILE Estimates with Other Published Studies</u>

We compared our heritability estimates for PheWAS code-based phenotypes to the heritability estimates from several previously published phenome-wide studies:

1. ***MS1:*** An independent study also using the MarketScan database [9], but analyzed without modeling shared community-level environment;

2. ***NY***: A study that repurposed EHR data from New York State [10]
3. ***CaTCH:*** A study [11] that analyzed twins from EHR data to estimate genetic and environmental contributions, and
4. ***LDSC-UKB***: Heritability estimates based upon GWAS summary statistics from UK Biobank [12].

The MS1 study estimated heritability and genetic correlations also in MarketScan database for 149 common diseases using 481,657 individuals grouped into 129,989 families. The *CaTCH* study estimated heritability in 55,396 twin pairs from claims data provided by Aetna, Inc. health insurance company. Notably, the *CaTCH* twin cohort is considerably younger than our cohort with age IQR being (3, 13). The *NY* study estimated heritability for groups of ICD-9/10 codes from family pedigrees constructed using emergency contact data at 3 medical centers in the state of New York. The *LDSC-UKB* study computed heritability for ICD-10 code groups by applying LD-score regression to GWAS summary statistics computed on traits from the UK-Biobank sample [13].

Correlations of heritability estimates between different studies are summarized in Table 2. Correlation with the *MS1* study was high, which is reassuring as both studies use the MarketScan database. Correlation was lowest in the *CaTCH* study, which is possibly due to the younger ages of the *CaTCH* twins. Individuals in *CaTCH* may be too young to have developed symptoms for many analyzed diseases. Another potential source of differences is the smaller sample size in *CaTCH*. For both *CaTCH* and the *LDSC-UKB* study, correlation was nearly doubled if only considering more common traits with prevalence > 1%.

We found SMILE generally yielded smaller estimates of heritability than GPC and the family-based studies, i.e., *NY*, *MS1*, and *CaTCH*. This is consistent with our simulation results, indicating shared community-level environmental risk, when left unaccounted for, could add to the upward bias of heritability estimates from family-based studies.

4. Robustness Analyses

a. Impact of the length of enrollment on heritability estimates.

We investigated whether the length of enrollment of study participants (6-12 years) may influence our phenotype definitions and the estimates of variance components in the SMILE model. To assess robustness, we reran models estimating variance components comparing families enrolled for 6-7 years only (149,710 families) to families enrolled for 10-12 years (39,247 families) for all 1,083 phenotypes. Models converged for both cohorts in 973/1083 phenotypes. For models that failed to converge, median phenotype prevalence is less than 0.003. Variance components estimates and standard errors for all converged models using families enrolled for 6-7 years and for 10-12 years may be found in Supplementary Data 4.

For the 447 phenotypes with prevalence >1% analyzed using families enrolled for 6-7 years and for 10-12 years, the average Pearson and Spearman correlation in genetic heritability estimates are 0.949 and 0.925 respectively. The 95% confidence intervals obtained using families with different enrollment lengths overlap for 88.4% of the phenotypes (Supplementary Figure 7). Our results suggest that length of enrollment has a minimal impact on heritability estimates in MarketScan.

b. Impact of drug code on phenotype definition and heritability estimates.

Phenotype definitions based only on primary provider diagnostic codes may be noisy, as they may fail to capture drug prescriptions provided through secondary providers and thus underestimate disease prevalence. To examine robustness of heritability estimation, we reanalyze Type 2 Diabetes (T2D) using phenotype definitions with both diagnosis and prescription information, as previously defined [14]. Specifically, we defined T2D cases to include patients taking any T2D drug prescriptions based on TriNetX or Food and Drug Administration (FDA) T2D drug prescription list, as well as any of the ICD diagnostic codes originally used to define T2D cases. Due to the limitation of MarketScan EHR data, the source of the prescriptions (primary vs. secondary care) was not available and therefore was not used in our phenotype definitions. Using this definition, T2D prevalence slightly increased from 9.2% to 9.99%. Both prevalence estimates are concordant with the Centers for Disease Control and

Prevention's (CDC) estimate of 9-9.5% (https://www.cdc.gov/diabetes/basics/type2.html). Of the original 9.2% of patients with T2D diagnostic codes, 51.8% had a T2D drug code. Of all patients who had T2D drug codes, only 0.9% did not have a corresponding diagnostic code. Using the new T2D phenotype definition, the heritability estimates increased from 0.284 (SE=0.009) to 0.31 (SE=0.008). Importantly, using this new phenotype definition, adding spatial random effect to the model continues to yield smaller heritability estimates compared to models without spatial random effects (heritability = 0.39, SE=0.008), supporting our main conclusion. Our analysis results with and without using drug code in the phenotype definition are shown in Supplementary Data 5. The full list of NDC drug codes used to define T2D cases is provided in Supplementary Data 6.

c. Impact of mis-specified pedigrees and heritability estimates.

It is important to assess the robustness of heritability estimates if the inferred relationship is not accurate. To evaluate the impact of relationship error on heritability estimates, we simulated binary phenotypes under a liability threshold model (See Variance Component Model Simulation section for more details) for 250,000 nuclear families of which 2.4% had at least one adopted child and 6.2% had at least one stepchild. Adopted child is assumed to be biologically unrelated to the parents and a stepchild is assumed to be biologically related to a randomly chosen parent in the family. Below, we provide a few examples of kinships matrix when there is adoptive or stepchild in the family. As a first example, the genetic relationship matrix used for families with 2 adoptive children is

$$\mathrm{G}_{\mathrm{adoptive}} = \begin{pmatrix} 1 & 0 & 0 & 0 \\ 0 & 1 & 0 & 0 \\ 0 & 0 & 1 & 0 \\ 0 & 0 & 0 & 1 \end{pmatrix}.$$

As a second example, we consider a family where each parent has a child from their previous marriage. The genetic relationship matrix used for families with 2 stepchildren, with the first child being the biological child of parent 2 and the second child being the biological child of parent 1, is

$$\mathrm{G}_{\mathrm{stepchildren}} = \begin{pmatrix} 1 & 0 & 0 & 0.5 \\ 0 & 1 & 0.5 & 0 \\ 0 & 0.5 & 1 & 0 \\ 0.5 & 0 & 0 & 1 \end{pmatrix}.$$

We simulated 20 replicates. For each replicate, the variance parameters are randomly simulated based on $\sigma_s^2 \sim \mathrm{unif}(0.01, 0.05)$, $\sigma_g^2 \sim \mathrm{unif}(0.3, 0.5)$, $\sigma_{\mathrm{par}}^2 \sim \mathrm{unif}(0.05, 0.2)$, and $\sigma_{\mathrm{child}}^2 \sim \mathrm{unif}(0.05,0.2)$, reflecting the estimates from MarketScan (Supplementary Data 2). Family locations were also sampled with replacement from real MarketScan locations, so that the fraction of families from each location reflects the real distributions. After generating the underlying liabilities, we created binary phenotypes under the liability threshold model for population prevalence's of 0.1%, 1%, and 10%. We then fit SMILE (GPC+S) model using the true genetic relation matrix and using inferred genetic relation matrix (GPC+S noisy family), where each child was treated as biological child to both parents even when they are stepchild or adopted. We next compared the heritability estimates from SMILE models fitted to "GPC+S accurate pedigree" and "GPC+S noisy pedigree" to evaluate the effect of mis-specified pedigrees. We observe the mean squared error between the estimates using the correct and noisy relationship matrix are around 0.00037 (Supplementary Figure 5). Given that the average length of the 95% confidence interval for heritability estimates is 0.032, the bias induced by relationship error is negligible (Supplementary Data 3).

d. Impact of pollution and wind measurement errors on pollution causal effect estimates.
As all other measurements, pollution and wind may also contain errors. Thus, it is important to assess how the causal effects of pollution may be affected if the pollution or wind measures are noisy. To do so, we performed simulations (similarly to Supplementary Text Section 9) to assess how noisy wind and pollution measurements may impact the type-1 error, power, and mean squared error (MSE) of estimated causal effects.

We use P_{true} to denote true pollution levels and $P_{observed}$ to denote observed pollution levels, which may contain errors. The originally measured pollution and wind are used as true values, which we denote as P_{true}. To generate realistic pollution and wind measures with noise, for each location, we sample from 10 neighbors and use the sampled values as noisy measures, which we denote as $P_{observed}$. Wind speed and directions with noise are also simulated using resampling as for pollution levels. We then simulate the disease liability as following:

$$y_{true} \sim f\left(P_{true}, X_{CLERF}, \boldsymbol{u_g}, \boldsymbol{u_{par}}, \boldsymbol{u_{child}}\right),$$

where X_{CLERF} is a matrix of geographical specific covariates (e.g., social economic status, average temperature, etc.), $\boldsymbol{u_g}$ is a vector of genetic random effects, $\boldsymbol{u_{par}}$ and $\boldsymbol{u_{child}}$ are vectors of random effects for the shared parental- and children-level family environment.

We considered simulation scenarios where pollution level is measured with error, where wind is measured with error, and where pollution and wind are measured without error. A total of 2500 replicates were simulated for each scenario.

We then analyze simulated y_{true} and noisy pollution and wind measures *(i.e.,* $P_{observed}$ and $W_{observed}$) in SMILE-2 as described in Supplementary Text Section 9 and the IND-FE model. We did not observe any inflation or difference in the Type-1 Error rates between the three SMILE-2 simulations (Supplementary Figure 6A). We observed 3.17% and 3.59% decrease in power and 3.99% and 5.93% increase in MSE when noisy pollution and wind measurements are used. However, the 95% confidence intervals of power and MSE using noisy pollution and wind measurements overlapped the 95% confidence interval based on observed pollution and wind measurements (Supplementary Figure 6A,B). Importantly, SMILE-2 model was still more powerful and had lower MSE when compared to IND-FE models under scenarios with noisy pollution and wind measurements, supporting our main conclusion (Supplementary Figure 6A,B). Together, our simulation study suggests that pollution causal effect inference by SMILE-2 remains unbiased and robust to measurement errors in pollution levels, and wind speed and directions.

5. Fitting SMILE Models via Laplace Approximation

We seek to fit the mixed effect models via Laplace approximation. We make extensive use of the R package TMB (13) for model estimation, which relies on the Automatic Differentiation (AD) software to calculate the gradients of the approximate likelihood. Specifically, for a SMILE model with GPC+S variance components, we let $\boldsymbol{\theta} = \left(\sigma^2_{par}, \sigma^2_{child}, \sigma^2_g, \sigma^2_s, \rho_s\right)$ denote the variance parameters of interest. We further denote the full random effects vector by $\boldsymbol{u} = (\boldsymbol{u}^{\boldsymbol{T}}_{\boldsymbol{par}}, \boldsymbol{u}^{\boldsymbol{T}}_{\boldsymbol{child}}, \boldsymbol{u}^{\boldsymbol{T}}_{\boldsymbol{g}}, \boldsymbol{u}^{\boldsymbol{T}}_{\boldsymbol{s}})^T$. We use N_F to denote total number of nuclear families and L to denote the total number of MarketScan locations. The random effect $\boldsymbol{u}$ follows a multivariate normal distribution:

$$f(\boldsymbol{u}|\boldsymbol{\theta}) \sim MVN(0, \textbf{blk\_diag}(\sigma^2_{par}\boldsymbol{I_{3F}}, \sigma^2_{child}\boldsymbol{I_{3F}}, \sigma^2_g\boldsymbol{G_{ALL}}, \boldsymbol{\Sigma_{S_{(L\times L)}}})$$

blk-diag denotes block-diagonal matrices, with blocks being $\sigma^2_{par}\boldsymbol{I_{3F}}, \sigma^2_{child}\boldsymbol{I_{3F}}, \sigma^2_g\boldsymbol{G_{ALL}}$ and $\boldsymbol{\Sigma_{S_{(L\times L)}}}$. $\boldsymbol{G_{ALL}}$ is the block diagonal kinship matrix for the entire dataset, i.e., $\boldsymbol{G_{ALL}} = blk\_diag\left(\boldsymbol{G_1}, \ldots, \boldsymbol{G_{N_F}}\right)$ where G_f is the genetic kinship for family f. Families are assumed to be independent of each other. In the example of a quad-family, the kinship is given by

$$\boldsymbol{G_f} = \begin{pmatrix} 1 & 0 & 0.5 & 0.5 \\ 0 & 1 & 0.5 & 0.5 \\ 0.5 & 0.5 & 1 & 0.5 \\ 0.5 & 0.5 & 0.5 & 1 \end{pmatrix}$$

The log likelihood for case-control status conditional on the random effects follows

$$f(\boldsymbol{Y}|\boldsymbol{X}, \boldsymbol{\beta}, \boldsymbol{u}) \quad \sim MVN(\boldsymbol{X\beta} + \boldsymbol{Z_{par}}\boldsymbol{u_{par}} + \boldsymbol{Z_{child}}\boldsymbol{u_{child}} + \boldsymbol{u_g} + \boldsymbol{Z_s}\boldsymbol{u_s}, \sigma^2_\epsilon I_{4F})$$

Our goal is to find the parameters $\boldsymbol{\theta}$ that maximize the marginal likelihood of disease status, i.e.,

$$L(\boldsymbol{Y}|\boldsymbol{X},\boldsymbol{\beta},\boldsymbol{\theta}) = \int \exp(l(\boldsymbol{Y}|\boldsymbol{X},\boldsymbol{\beta},\boldsymbol{u}) + l(\mathbf{u}|\boldsymbol{\theta}))\, d\boldsymbol{u}$$

where we integrate out the random effects $\boldsymbol{u}$ from the joint likelihood.

We use $\boldsymbol{u}^*$ to denote the solution which maximizes the joint log-likelihood function given a fixed value of $\boldsymbol{\theta}$ and $\boldsymbol{\beta}$, i.e.

$$\boldsymbol{u}^*(\boldsymbol{\theta},\boldsymbol{\beta}) = argmax_{\mathbf{u}} l(\boldsymbol{Y};\boldsymbol{X},\boldsymbol{\beta},\boldsymbol{\theta},\boldsymbol{u})$$

Further we let $H(\boldsymbol{\theta},\boldsymbol{\beta})$ denote the Hessian matrix of the joint density $f(\boldsymbol{Y};\boldsymbol{X},\boldsymbol{\beta},\mathbf{u},\boldsymbol{\theta})$ with respect to random effects $\boldsymbol{u}$ and evaluated at $\boldsymbol{u}^*(\boldsymbol{\theta},\boldsymbol{\beta})$ by

$$H(\boldsymbol{\theta},\boldsymbol{\beta}) \quad = l''_{uu}(\boldsymbol{u}^*(\boldsymbol{\theta},\boldsymbol{\beta}),\boldsymbol{\theta})|_{\mathbf{u}=\mathbf{u}^*}$$

A Taylor series expansion of $\boldsymbol{u}$ around $\boldsymbol{u}^*$ gives the approximation

$$\tilde{l}(\boldsymbol{Y};\boldsymbol{X},\boldsymbol{\theta},\boldsymbol{\beta},\boldsymbol{u}) \quad \approx l(\boldsymbol{Y}|\boldsymbol{X},\boldsymbol{\theta},\boldsymbol{\beta},\boldsymbol{u}^*) - \frac{1}{2}(\boldsymbol{u}-\boldsymbol{u}^*)^T H(\boldsymbol{\theta},\boldsymbol{\beta})(\boldsymbol{u}-\boldsymbol{u}^*)$$

We will use the approximation $\tilde{l}$ in the integrand to calculate the marginal likelihood, which equals to:

$$\begin{aligned} L^*(\theta) \quad &= log\left[\int_{\mathbb{R}^{10F+L}} \exp(l(\boldsymbol{Y}|\boldsymbol{X},\boldsymbol{\theta},\boldsymbol{\beta},\boldsymbol{u}^*) - \frac{1}{2}(\boldsymbol{u}-\boldsymbol{u}^*)^T H(\boldsymbol{\theta},\boldsymbol{\beta})(\boldsymbol{u}-\boldsymbol{u}^*))\, d\boldsymbol{u}\right] \\ &= l(\boldsymbol{Y}|\boldsymbol{X},\boldsymbol{\theta},\boldsymbol{\beta},\boldsymbol{u}^*)) + \frac{\dim(\boldsymbol{u})}{2} log(2\pi) - \frac{1}{2} log(det(H(\boldsymbol{\theta},\boldsymbol{\beta}))) \end{aligned}$$

The approximate likelihood can be maximized using penalized least square method and standard nonlinear optimization algorithms.

6. Conversion to Liability Scale Variance Components

After estimating variance components on the observed scale for a phenotype using the SMILE model, we convert the variance components to the liability scale. Liability scale variance components for binary traits allows for the comparison of heritability and environmental variance estimates between diseases with different prevalence[15].

Using the liability threshold model [16], we assume that that there is a continuous underlying score l for binary disease traits which follows a standard normal distribution

$$l \sim N(0,1)$$

We further assume that the observed disease status $y \in \{0,1\}$ is determined by whether the liability l surpasses a threshold T as determined by population disease prevalence K, i.e.,

$$T = \Phi^{-1}(1-K)$$

$$y = \begin{cases} 1 & l > T \\ 0 & l \leq T \end{cases}$$

where Φ is the cumulative distribution function for a standard normal random variable. It can be shown that the expectation and variance of the liability for diseased individuals is

$$\begin{aligned} E(l|l > T) = &\quad a = \phi(T)/K \\ V(l|l > T) &\quad = 1 - a(a-T) \end{aligned}$$

The basic idea for the conversion formula is to link the estimate of the recurrence risk for two family members on the observed scale with that on the liability scale.

First let l_1 and l_2 denote the liability of two relatives within a family, then it can be shown [17] that

$$
\begin{aligned}
E(l_2|l_1 > T) &= ar_{lia} \\
V(l_2|l_1 > T) &= 1 - r_{lia}^2(a(a-T))
\end{aligned}
$$

where r_{lia} is the covariance of the liabilities between relatives. Reich et al. [18] modified Falconer's formula [16] to account for decreased variance in the liability distribution among relatives. If we standardize the distribution of relatives of diseased individuals such that their liability follows an approximate standard normal distribution, i.e., $l_2 \sim N(0,1)$, then the recurrence risk is

$$K_R = E(I(l_2 > T) = 1|l_1 > T) = \Pr(I(l_2 > T) = 1|l_1 > T) = 1 - \Phi(T_R) \qquad (S1)$$

where T_R is given by:

$$T_R = \frac{T - ar_{lia}}{\sqrt{1 - r_{lia}^2\big(a(a-T)\big)}} \qquad (S2)$$

From (S2), we can solve for r_{lia}

$$r_{lia} = \frac{T - T_R\sqrt{1 - (T^2 - T_R^2)(1 - T/a)}}{a + T_R^2(a-T)} \qquad (S3)$$

We also seek to estimate the recurrence risk K_R on the observed scale. We use y_1 and y_2 to denote the observed phenotypes for a pair of relatives within a family. They satisfy:

$$
\begin{aligned}
E(y_1) = E(y_2) &= K \\
E(y_2|y_1 = 1) &= K_R
\end{aligned}
$$

The covariance of the observed phenotypes can also be written as a function of the recurrence risk:

$$
\begin{aligned}
r_{obs} &= Cov(y_1, y_2) \\
&= E(y_1 y_2) - E(y_1)E(y_2) \\
&= E(y_2|y_1 = 1)P(y_1 = 1) - E(y_1)E(y_2) \\
&= KK_R - K^2
\end{aligned} \qquad (S4)
$$

From (S3), we can also derive recurrence risk as a function of observed phenotypic covariance between relatives:

$$
\begin{aligned}
K_R &= K + \frac{r_{obs}}{K} \\
T_R &= \Phi^{-1}(1 - K_R)
\end{aligned} \qquad (S5)
$$

Using equations (S2) and (S4), we will be able to convert phenotypic covariances in observed scale to phenotypic covariances in liability scale. Plugging the observed scale covariance estimate r_{obs} into (S5), solving for T_R, and plugging T_R into (S3), we can express r_{lia} as a function of r_{obs}.

Finally, as observed and liability scale phenotypic covariances are functions of the variance components, we will be able to convert variance component estimates between observed scale and liability scale. Specifically, conditioning on relevant individual level fixed effects, the covariances of observed phenotypes are equal to:

$$
\begin{aligned}
r_{obs,par-par} &= \tilde{\sigma}_{s,obs}^2 + \sigma_{par,obs}^2 \\
r_{obs,child-child} &= \tilde{\sigma}_{s,obs}^2 + \frac{1}{2}\sigma_{g,obs}^2 + \sigma_{child,obs}^2 \\
r_{obs,par-child} &= \tilde{\sigma}_{s,obs}^2 + \frac{1}{2}\sigma_{g,obs}^2 \\
r_{obs,s-s} &= \tilde{\sigma}_{s,obs}^2
\end{aligned} \qquad \text{(S6)}
$$

where $par-par$,$child-child$, and $par-child$ indicate relationships between two parents, two children, and a parent and a child within the same family. The covariances between individuals due to shared community-level environment is denoted as $s-s$.

As the covariances of observed phenotypes can be converted to the covariances between liabilities, we can obtain liability scale variance components using (S6) and below:

$$\begin{aligned} \sigma^2_{g,lia} &= 2(r_{lia,par-child} - r_{lia,s-s}) \\ \sigma^2_{par,lia} &= r_{lia,par-child} - r_{lia,s-s} \\ \sigma^2_{sib,lia} &= r_{lia,child-child} - r_{lia,par-child} \\ \tilde{\sigma}^2_{s,lia} &= r_{lia,s-s} \end{aligned} \tag{S7}$$

7. Standard Errors for Liability-scale Variance Components by Multivariate Delta Method

We describe the calculation of standard errors for variance components estimates in SMILE model. Let $\hat{\boldsymbol{\theta}}_{\boldsymbol{obs}} = (\sigma^2_{g,obs}, \sigma^2_{par,obs}, \sigma^2_{child,obs}, \sigma^2_{s,obs}, \rho)^T$ denote the maximum likelihood estimates of variance parameters from the full SMILE model, which is fitted to observed case control status. The covariance matrix of $\hat{\boldsymbol{\theta}}_{\boldsymbol{obs}}$ is given by the inverse of the Fisher information matrix evaluated at the maximum likelihood estimates $\hat{\boldsymbol{\theta}}_{\boldsymbol{obs}}$, i.e.,

$$V(\hat{\boldsymbol{\theta}}_{\boldsymbol{obs}}) = -\nabla_{\theta_{obs}} \nabla^T_{\theta_{obs}} l(\boldsymbol{\theta}_{\boldsymbol{obs}})|_{\theta_{obs}=\hat{\theta}_{obs}}$$

The maximum likelihood estimates asymptotically satisfy:

$$(\hat{\boldsymbol{\theta}}_{\boldsymbol{obs}} - \boldsymbol{\theta}_{\boldsymbol{obs}}) \xrightarrow{d} MVN(0, V(\hat{\boldsymbol{\theta}}_{\boldsymbol{obs}}))$$

As the liability scale variance components may be transformed from observed scale estimates, the variance-covariance matrix of the liability scale estimates can be derived using multivariate delta method. We denote the transformation from observed to liability scale estimates as

$$\boldsymbol{g}(\boldsymbol{\theta}_{\boldsymbol{obs}}) = \boldsymbol{\theta}_{\boldsymbol{lia}} = (\sigma^2_{g,lia}, \sigma^2_{par,lia}, \sigma^2_{child,lia}, \tilde{\sigma}^2_{s,lia})^T$$

The function $\boldsymbol{g}$ can be broken down into the compositions of the following steps:

$$\begin{aligned} \boldsymbol{\theta}_{\boldsymbol{obs}} &= (\sigma^2_{g,obs}, \sigma^2_{par,obs}, \sigma^2_{child,obs}, \sigma^2_{s,obs}, \rho)^T \\ g_0(\boldsymbol{\theta}_{\boldsymbol{obs}}) &= (\sigma^2_{g,obs}, \sigma^2_{par,obs}, \sigma^2_{child,obs}, \tilde{\sigma}^2_{s,obs})^T \\ g_1(g_0(\boldsymbol{\theta}_{\boldsymbol{obs}})) &= (r_{parent-parent,obs}, r_{parent-child,obs}, r_{child-child,obs}, r_{s,obs})^T \\ g_2(g_1(g_0(\boldsymbol{\theta}_{\boldsymbol{obs}}))) &= (r_{parent-parent,lia}, r_{parent-child,lia}, r_{child-child,lia}, r_{s,lia})^T \\ \mathbf{g}(\boldsymbol{\theta}_{\boldsymbol{obs}}) = g_3(g_2(g_1(g_0(\boldsymbol{\theta}_{\boldsymbol{obs}})))) &= (\sigma^2_{g,lia}, \sigma^2_{par,lia}, \sigma^2_{child,lia}, \tilde{\sigma}^2_{s,lia})^T \end{aligned} \tag{S8}$$

Individually,

- g_0 transforms $\sigma^2_{s,obs}$ and ρ to the Gower factor for the spatial variance component $\tilde{\sigma}^2_{s,obs}$.
- g_1 transforms observed scale variance components to observed scale family covariances, as in equation (S6).
- g_2 transforms covariances of observed phenotypes to the covariances of liabilities between family members.
- g_3 transforms covariances of liabilities to liability scale variance components, as in equation (S7).

To obtain standard errors for $\boldsymbol{g}$, we successively apply the multivariate delta method:

$$
\begin{aligned}
g_0(\widehat{\boldsymbol{\theta}}_{\boldsymbol{obs}}) &\overset{d}{\to} MVN(g_0(\boldsymbol{\theta}_{\boldsymbol{obs}}), \nabla g_0 V(\widehat{\boldsymbol{\theta}}_{\boldsymbol{obs}}) \nabla g_0^T) \\
g_1(g_0(\widehat{\boldsymbol{\theta}}_{\boldsymbol{obs}})) &\overset{d}{\to} MVN(g_1(g_0(\boldsymbol{\theta}_{\boldsymbol{obs}})), \nabla g_1 \nabla g_0 V(\widehat{\boldsymbol{\theta}}_{\boldsymbol{obs}}) \nabla g_0^T \nabla g_1^T) \\
&\vdots \\
\mathbf{g}(\widehat{\boldsymbol{\theta}}_{\boldsymbol{obs}}) &\overset{d}{\to} MVN(g(\boldsymbol{\theta}_{\boldsymbol{obs}}), \nabla g_3 \nabla g_2 \nabla g_1 \nabla g_0 V(\widehat{\boldsymbol{\theta}}_{\boldsymbol{obs}}) \nabla g_0^T \nabla g_1^T \nabla g_2^T \nabla g_3^T)
\end{aligned}
$$

Here, we use ∇g_i to denote the derivative of g_i with respect to its immediate input, e.g.,

$$\nabla_{g_2}\left(g_2\left(g_1(g_0(\theta))\right)\right) = g_2'\left(g_1(g_0(\theta))\right)$$

To begin, we can describe in detail the first transformation g_0. For notational convenience, we rewrite the spatial covariance matrix as $\boldsymbol{\Sigma_S} = \sigma_s^2 \boldsymbol{\Omega_S}$. For CAR and SAR models, we have

$$
\begin{aligned}
\boldsymbol{\Omega}_{\mathbf{S,SAR}} &= ((I - \rho \boldsymbol{M}^{-\frac{1}{2}} \boldsymbol{W}_+ \boldsymbol{M}^{\frac{1}{2}}) M^{-1} (I - \rho \boldsymbol{M}^{-\frac{1}{2}} \boldsymbol{W}_+ \boldsymbol{M}^{\frac{1}{2}}))^{-1} \\
\boldsymbol{\Omega}_{\mathbf{S,CAR}} &= (\boldsymbol{M}^{-\frac{1}{2}} (I - \rho \boldsymbol{M}^{-\frac{1}{2}} \boldsymbol{W}_+ \boldsymbol{M}^{\frac{1}{2}}) \boldsymbol{M}^{-\frac{1}{2}})^{-1}
\end{aligned}
$$

where $\boldsymbol{W}$ is a matrix of indicators for neighboring locations where the i,j^{th} element is 1 if locations i and j share a geographical border and 0 otherwise and

$$\boldsymbol{M} = diag\left(\left(\sum_j W_{1j}\right)^{-1}, \dots, \left(\sum_j W_j\right)^{-1}\right)$$

$$\boldsymbol{W}_+ = \boldsymbol{MW}$$

Our community-level spatial variance component $\tilde{\sigma}_s^2$ is a function of the two random variables ρ and $\sigma_{s,obs}^2$, which describes the amount of phenotypic variance explained by the spatial random effect.

$$
\begin{aligned}
f(\sigma_s^2, \rho) = \tilde{\sigma}_s^2 &= \frac{\sigma_s^2}{N} tr(\boldsymbol{Z}(I - \frac{1}{L}\mathbf{1}\mathbf{1}^T)\boldsymbol{\Omega_S}\boldsymbol{Z}^T) \\
\nabla_{\sigma_s^2} f &= \frac{1}{N} tr(\boldsymbol{Z}(\boldsymbol{I} - \frac{1}{L}\mathbf{1}\mathbf{1}^{\boldsymbol{T}})\boldsymbol{\Omega_S}\boldsymbol{Z}^{\boldsymbol{T}}) \\
\nabla_\rho f &= \sigma_s^2 (I - \frac{1}{L}\mathbf{1}\mathbf{1}^{\boldsymbol{T}})\boldsymbol{Z}^{\boldsymbol{T}}\boldsymbol{Z}(\nabla_\rho \boldsymbol{\Omega_S})
\end{aligned}
$$

Where for CAR and SAR models we have

$$
\begin{aligned}
\nabla_\rho \Omega_{S,SAR} &= -\boldsymbol{\Omega_S}(2M^{-1}(I - \rho \boldsymbol{M}^{-\frac{1}{2}} \boldsymbol{W}_+ \boldsymbol{M}^{\frac{1}{2}}))\boldsymbol{\Omega_S} \\
\nabla_\rho \Omega_{S,CAR} &= -\boldsymbol{\Omega_S}(\boldsymbol{M}^{-\frac{3}{2}} \boldsymbol{W}_+ \boldsymbol{M}^{\frac{1}{2}})\boldsymbol{\Omega_S}
\end{aligned}
$$

We then apply the delta method to obtain standard errors for **g** using the gradients for g_0,g_1,g_2, g_3 given by:

$$
\nabla_{g_0} = \begin{array}{c} \\ \sigma_{par,obs}^2 \\ \sigma_{child,obs}^2 \\ \sigma_{s,obs}^2 \end{array}
\begin{array}{ccccc} \sigma_{g,obs}^2 & \sigma_{par,obs}^2 & \sigma_{child,obs}^2 & \sigma_{s,obs}^2 & \rho \\ 0 & 1 & 0 & 0 & 0 \\ 0 & 0 & 1 & 0 & 0 \\ 0 & 0 & 0 & \nabla_{\sigma_s^2} f & \nabla_\rho f \end{array}
$$

and

$$
\sigma_{g,obs}^2 \quad \sigma_{par,obs}^2 \quad \sigma_{child,obs}^2 \quad \sigma_{s,obs}^2
$$

$$\nabla_{g_1} = \begin{array}{r} r_{par-par,obs} \\ r_{par-child,obs} \\ r_{child-child,obs} \\ r_{s-s,obs} \end{array} \begin{bmatrix} 0 & 1 & 0 & 1 \\ \frac{1}{2} & 0 & 0 & 1 \\ \frac{1}{2} & 0 & 1 & 1 \\ 0 & 0 & 0 & 1 \end{bmatrix}$$

Function g_2 transforms the covariances of observed phenotypes to the covariances of liabilities as in (S4) and (S5). For a particular family relationship ι, (i.e., ι can be par-par, par-child, or child-child, or s-s (same community location)), we let

$$\begin{aligned}
\frac{\partial r_{lia,\iota}}{\partial r_{obs,\iota}} &= \psi_\iota = \frac{\partial r_{lia,\iota}}{\partial T_{R,\iota}} \frac{\partial T_{R,\iota}}{\partial K_{R,\iota}} \frac{\partial K_{R,\iota}}{\partial r_{obs,\iota}} \\
\frac{\partial r_{lia,\iota}}{\partial T_{R,\iota}} &= \frac{-\sqrt{1-(T^2-T_{R,\iota}^2)(1-T/a)(1+T_{R,\iota}^2(a-T))})}{a+T_{R,\iota}^2(a-T)} \\
&\quad - \frac{2T_{R,\iota}(a-T)(T-T_{R,\iota}\sqrt{1-(T^2-T_{R,\iota}^2)(1-T/a)})}{(a+T_{R,\iota}^2(a-T))^2} \\
\frac{\partial T_R}{\partial K_{R,\iota}} &= -\frac{1}{\phi(\Phi^{-1}(1-K_{R,\iota}))} \\
\frac{\partial K_{R,\iota}}{\partial r_{obs,\iota}} &= \frac{1}{K}
\end{aligned}$$

Then the derivatives for the transformation g_2 is equal to:

$$\nabla_{g_2} = \begin{array}{r} \\ r_{par-par,lia} \\ r_{par-child,lia} \\ r_{child-child,lia} \\ r_{s-s,lia} \end{array} \begin{array}{cccc} r_{par-par,obs} & r_{par-child,obs} & r_{child-child,obs} & r_{s-s,obs} \\ \psi_{par-par} & 0 & 0 & 0 \\ 0 & \psi_{child-child} & 0 & 0 \\ 0 & 0 & \psi_{par-child} & 0 \\ 0 & 0 & 0 & \psi_{s-s} \end{array}$$

And finally for g_3, which transforms the covariances of liabilities to liability scale variance components as in (S7), its derivative is given by:

$$\nabla_{g_3} = \begin{array}{r} \\ \sigma_{g,lia} \\ \sigma^2_{par,lia} \\ \sigma^2_{child,lia} \\ \tilde{\sigma}^2_{s,lia} \end{array} \begin{array}{cccc} r_{par-par,lia} & r_{par-child,lia} & r_{child-child,lia} & r_{s-s,lia} \\ 0 & 2 & 0 & -2 \\ 1 & 0 & 0 & -1 \\ 0 & -1 & 1 & 0 \\ 0 & 0 & 0 & 1 \end{array}$$

Plugging gradients in to (S8) returns the standard errors for liability scale variance components.

8. Converting relative risk in observed scale to effects in liability scale

In our simulations, we simulated liabilities of binary diseases, dichotomized the liability based on the disease prevalence, and obtained binary phenotypes. In order to understand the average liability scale covariate effects for binary disease outcomes, we derive formulae to convert them to odds ratios. The key idea for this conversion is to calculate average relative risks in liability and observed scale for the covariates and use them to derive relationships between covariate effects on the liability and binary disease status.

Under the observed scale, linear regression is used where the 0-1 disease status is modeled as a linear function of the covariates and random effects, i.e.,

$$\boldsymbol{Y} = \widetilde{\boldsymbol{P}}\boldsymbol{\beta} + \boldsymbol{X}\boldsymbol{\pi}_2 + \boldsymbol{u}_g + \boldsymbol{Z}_{par}\boldsymbol{u}_{par} + \boldsymbol{Z}_{child}\boldsymbol{u}_{child} + \boldsymbol{\epsilon}$$

where all covariates were mean-centered and scaled to have standard deviation of 1 prior to initial model fitting.

We let the superscript '$*$' denote the corresponding parameter effects on the liability-scale. The liability l is modeled as a function of fixed and random effects, i.e.,

$$\boldsymbol{l} = \widetilde{\boldsymbol{P}}\boldsymbol{\beta}^* + \boldsymbol{X}\boldsymbol{\pi}_2^* + \boldsymbol{u}_g^* + \boldsymbol{Z}_{par}\boldsymbol{u}_{par}^* + \boldsymbol{Z}_{child}\boldsymbol{u}_{child}^* + \boldsymbol{\epsilon}^*$$

We denote the standard deviation of observed scale cumulative confounding effect as

$$b = sd(\boldsymbol{X}\boldsymbol{\pi}_2)$$

which we use as a measure of the unit of change in the cumulative confounding effect.

The relative risk of having one unit of increase in the cumulative confounding effect over the baseline is equal to

$$RR = \frac{\Pr\left(Y_i = 1 \middle| X_i^T\pi_2 = b\right)}{\Pr\left(Y_i = 1 \middle| X_i^T\pi_2 = 0\right)} = \frac{E\left(Y_i \middle| X_i^T\pi_2 = b\right)}{E\left(Y_i \middle| X_i^T\pi_2 = 0\right)} = \frac{K + b}{K} \quad \text{(S9)}$$

Using the estimated relative risk associated with b, we can solve for the average liability scale effect b^*. Under the liability threshold scale, the relative risk given one unit of change in the liability effect can be expressed as:

$$RR = \frac{\Pr\left(Y_i = 1 \middle| X_i^T\pi_2^* = b^*\right)}{\Pr\left(Y_i = 1 \middle| X_i^T\pi_2^* = 0\right)} = \frac{\Pr\left(l_i > T \middle| X_i^T\pi_2^* = b^*\right)}{\Pr\left(l_i > T \middle| X_i^T\pi_2^* = 0\right)} = \frac{1 - \Phi(T - b^*)}{1 - \Phi(T)} \quad (S10)$$

Combining (S9) and (S10), we will be able to solve b^* as a function of b. We then rescale our confounding predictor effects used in the simulation to match the liability scale average effect.

$$\pi_2^* \approx \pi_2\left(\frac{b^*}{b}\right)$$

We note that this transformation is not exact but can serve as a useful approximation to link liability and observed scale estimates of covariate effects.

9. Two Stage Regression Model for Causal Inference of $PM_{2.5}$ and NO_2 Air Pollution

In addition to characterizing overall phenotypic variation explainable by spatial environment, we also leveraged two stage regression to assess the causality of individual environmental risk factors, including pollutants $PM_{2.5}$ and NO_2, which we collectively denote as $\boldsymbol{P}$. We incorporated environmental data from external sources and used wind speed and directions (as captured by sine and cosine of the vector of direction) as instrumental variables, which we collectively denote as $\boldsymbol{Z}$. Wind speed and direction have previously been used as instrumental variables for various pollution exposures [19-22]. Wind speed and direction are unlikely to have

any direct causal relationship with a disease phenotype, but are strong predictors of local pollution levels, as the pollution level in a given location is a mixture of both locally produced and transported air pollution carried by the wind from its original source [22, 23].

It is easy to verify that the wind speed and direction satisfy the three primary assumptions of instrumental variables in two-stage regression analyses[24]:

- Instrument relevance: the instrument is correlated with the pollutant $\boldsymbol{P}$, i.e., $cov(\boldsymbol{Z},\boldsymbol{P}) \neq 0$
- Instrument exogeneity: The instrument (averaged wind-direction) is uncorrelated with other confounders (measured or unmeasured) in the second stage model.
- The averaged wind-direction instruments $\boldsymbol{Z}$ have no direct effect on the disease phenotype $\boldsymbol{Y}$.

In the first stage model, the pollutant ($\boldsymbol{P}$) is regressed against the instrument of local averaged wind speed and direction ($\boldsymbol{Z}$), and a set of community-level environmental risk factor (CLERF) variables $\boldsymbol{X_{CLERF}}$ which include sociodemographic census variables and climate variables (i.e., averaged precipitation, and averaged monthly minimum and maximum temperature). CLERF variables may act as proxy for unmeasured confounding effects on the disease even if they do not have a direct interpretation by themselves. For example, population density may act as a proxy for noise pollution or urbanicity effects.

In order to incorporate instrumental variables in the first stage regression, we implement a strategy similar to Deryugina et al [22], where we constructed C clusters of the centroids of MarketScan locations by longitude and latitude using a k-means clustering algorithm. To choose the number of spatial clusters C, we fit the first stage model using different numbers of clusters (i.e., $C = 20, 25, \dots, 45, 50$) and selected the number of clusters that gives the largest adjusted R^2.

We then constructed a matrix of instruments linking each individual to the wind direction and speed at their MarketScan county/MSA location. Specifically, let W_l be a vector of length 3 containing the average wind speed, and cosine and sine of the averaged wind direction at location l, where $l = 1, \dots, L$. Further let $\boldsymbol{c}_l = (c_{l1}, \dots, c_{lC})^T$ be a $C \times 1$ indicator vector where element c_{lc} is 1 if location l is in cluster c and 0 otherwise. Then the matrix of instrumental variables can be represented as:

$$\boldsymbol{Z} = \begin{bmatrix} \boldsymbol{c}_1^T \otimes W_1^T \\ \boldsymbol{c}_2^T \otimes W_2^T \\ \vdots \\ \boldsymbol{c}_L^T \otimes W_L^T \end{bmatrix} \qquad (S11)$$

The first stage model takes the form of

$$\boldsymbol{P} = \boldsymbol{Z\gamma} + \boldsymbol{X_{CLERF}\pi_1} + \boldsymbol{u}$$

After estimating the first stage model, the pollution level is predicted based on the cluster-specific wind speeds and directions

$$\widetilde{\boldsymbol{P}} = \boldsymbol{Z}\,\widehat{\boldsymbol{\gamma}}$$

In the second stage regression, we make use of what we call the SMILE-2 model to assess the causal effect of pollution levels. The SMILE-2 model is specified as:

$$\boldsymbol{Y} = \widetilde{\mathbf{P}}\boldsymbol{\beta} + \mathbf{X}\boldsymbol{\pi_2} + \boldsymbol{u_g} + \mathbf{Z}_{\mathbf{par}}\boldsymbol{u_{par}} + \mathbf{Z}_{\mathbf{child}}\boldsymbol{u_{child}} + \boldsymbol{\epsilon} \qquad (S12)$$

SMILE-2 is analogous to the SMILE model, except that the focus is on estimating the causal effects of pollution on disease while controlling for genetic and family-level environmental effects. The spatial random effect is no longer incorporated as it has been partially explained by the air pollution level. Inclusion of spatial random effects will bias the inference of fixed effects [25, 26]. We used heteroscedastic-robust standard errors that were clustered at the location level to account for residual dependence within MarketScan locations. The detailed formula is described in the section below.

10. Clustered Robust Standard Errors for Causal Effect Estimates in SMILE-2 Model

Below, we show the derivations for the standard errors for the causal effect estimates in SMILE-2 model. We let N_F denote the number of nuclear family in the mixed model, N denote the total number of individuals in the

dataset, and L denote the number of MarketScan locations. Let X be a matrix of the q fixed effects covariates, with each column being a covariate, and P be a $4N_F \times p$ dimensional matrix of air pollutants (with $p \in (1,2)$). Then the marginal likelihood of case control status $\boldsymbol{Y}$ is

$$\boldsymbol{Y} \sim MVN(\widetilde{\boldsymbol{P}}\boldsymbol{\beta} + \boldsymbol{X}\boldsymbol{\pi}_2, \boldsymbol{V})$$

$$\boldsymbol{V} = \sigma_{par}^2 \boldsymbol{Z}_{par} \boldsymbol{Z}_{par}^T + \sigma_{child}^2 \boldsymbol{Z}_{child} \boldsymbol{Z}_{child}^T + \sigma_g^2 \boldsymbol{G}_{ALL}$$

The inference on $\boldsymbol{\beta}$ is of primary interest. We write the log-likelihood for $\boldsymbol{Y}$ as

$$l(\boldsymbol{\beta}, \boldsymbol{\pi}_2, \boldsymbol{\sigma}^2; \boldsymbol{Y}) = -\frac{n}{2} log(2\pi) - \frac{1}{2} log(|\boldsymbol{V}|) - \frac{1}{2} (\boldsymbol{Y} - \widetilde{\boldsymbol{P}}\boldsymbol{\beta} - \boldsymbol{X}\boldsymbol{\pi}_2)^T \boldsymbol{V}^{-1} (\boldsymbol{Y} - \boldsymbol{P}\boldsymbol{\beta} - \boldsymbol{X}\boldsymbol{\pi}_2)$$

where the gradient with respect to the fixed effect vector $(\boldsymbol{\beta}^T, \boldsymbol{\pi}_2^T)^T$ is given by

$$\nabla_{\beta,\pi_2} l(\boldsymbol{\beta}, \boldsymbol{\pi}_2, \boldsymbol{\sigma}^2) \quad = [\widetilde{\boldsymbol{P}}, \boldsymbol{X}]^T \boldsymbol{V}^{-1} (\boldsymbol{Y} - \widetilde{\boldsymbol{P}}\boldsymbol{\beta} - \boldsymbol{X}\boldsymbol{\pi}_2)$$

We write the score function as

$$s(\boldsymbol{\beta}, \boldsymbol{\pi}_2; \boldsymbol{y}) \quad = \left([\widetilde{\boldsymbol{P}}, \boldsymbol{X}]^T \boldsymbol{V}^{-1}\right)^T \circ (\boldsymbol{Y} - \widetilde{\boldsymbol{P}}\boldsymbol{\beta} - \boldsymbol{X}\boldsymbol{\pi}_2)$$

Where the '∘' operator denotes the Hadamard element-wise product. This score function is an $N \times (q + p)$ matrix, with each column corresponding to a fixed effect parameter and each row corresponding to an individual sample.

The Fisher information matrix for fixed effects is given by

$$-E(\nabla_{\beta,\pi_2} \nabla_{\beta,\pi_2}^T l(\boldsymbol{\beta}, \boldsymbol{\pi}_2, \boldsymbol{\sigma}^2) = [\widetilde{\boldsymbol{P}}, \boldsymbol{X}]^T \boldsymbol{V}^{-1} [\widetilde{\boldsymbol{P}}, \boldsymbol{X}]$$

Letting $\boldsymbol{A} = -E\left(\nabla_{\beta,\pi_2} \nabla_{\beta,\pi_2}^T l(\boldsymbol{\beta}, \boldsymbol{\pi}_2, \boldsymbol{\sigma}^2)\right)$ and $\boldsymbol{\psi} = cov(\nabla_{\beta,\pi_2} l(\boldsymbol{\beta}, \boldsymbol{\pi}_2, \boldsymbol{\sigma}^2))$, then the Huber-White sandwich estimator for covariance of $(\widehat{\boldsymbol{\beta}}^T, \widehat{\boldsymbol{\pi}}^T)$ takes the form

$$cov(\left[\widehat{\boldsymbol{\beta}}^T, \widehat{\boldsymbol{\pi}}_2{}^T\right]^{\mathrm{T}}) = \boldsymbol{A}^{-1} \boldsymbol{\psi} \boldsymbol{A}^{-1}$$

When samples are independent, $\boldsymbol{\psi} = \boldsymbol{A}$ and the covariance matrix $cov(\left[\widehat{\boldsymbol{\beta}}^T, \widehat{\boldsymbol{\pi}}^T\right]^T)$ reduces to the inverse of the Fisher information matrix.

Due to multiple families being nested within geographic locations, we cluster the standard errors at the MarketScan location-level, allowing for the possibility that model residuals may be correlated within county or MSA location. We calculate the matrix $\boldsymbol{\psi}$ empirically using

$$\boldsymbol{\psi} = \sum_{l=1}^{L} \left[\sum_{i \in C_l} s_i(\boldsymbol{\beta}, \boldsymbol{\pi}_2; \boldsymbol{Y})\right]^T \left[\sum_{i \in C_l} s_i(\boldsymbol{\beta}, \boldsymbol{\pi}_2; \boldsymbol{Y})\right]$$

where the summation of scores s_i is taken over all MarketScan individuals who live in the same geographic location c_l.

REFERENCE

1. Geddes, J.A., et al., *Long-term Trends Worldwide in Ambient NO2 Concentrations Inferred from Satellite Observations for Exposure Assessment.* Environmental Health Perspectives, 2016. **124**(3): p. 281-289.
2. Geddes, J.A., et al., *Global 3-Year Running Mean Ground-Level Nitrogen Dioxide (NO2) Grids from GOME, SCIAMACHY and GOME-2*. 2017, NASA Socioeconomic Data and Applications Center (SEDAC): Palisades, NY.
3. van Donkelaar, A., et al., *Global Annual PM2.5 Grids from MODIS, MISR and SeaWiFS Aerosol Optical Depth (AOD) with GWR, 1998-2016*. 2018, NASA Socioeconomic Data and Applications Center (SEDAC): Palisades, NY.
4. van Donkelaar, A., et al., *Global Estimates of Fine Particulate Matter Using a Combined Geophysical-Statistical Method with Information from Satellites.* Environmental Science & Technology, 2016. **50**(7): p. 3762.
5. Denny, J.C., et al., *Systematic comparison of phenome-wide association study of electronic medical record data and genome-wide association study data.* Nat Biotechnol, 2013. **31**(12): p. 1102-10.
6. Truven Health Analytics. *Commercial Claims and Encounters Medicare Supplemental*. 2016; Available from: *https://theclearcenter.org/wp-content/uploads/2020/01/IBM-MarketScan-User-Guide.pdf*.
7. U. S. Census Bureau, *American Community Survey 5-Year Estimates*, in *tidycensus: Load US Census Boundary and Attribute Data as 'tidyverse' and 'sf'-Ready Data Frames. R package version 0.9.9.5.* 2015: *https://CRAN.R-project.org/package=tidycensus*.
8. *U.S. Wind Climatology U-Component, V-Component, Mean Wind Speed Monthly Datasets*. National Oceanic and Atmospheric Administration: *ftp://ftp.cdc.noaa.gov/Datasets/ncep.reanalysis.dailyavgs/surface/*.
9. Wang, K., et al., *Classification of common human diseases derived from shared genetic and environmental determinants.* Nat Genet, 2017. **49**(9): p. 1319-1325.
10. Polubriaginof, F.C.G., et al., *Disease Heritability Inferred from Familial Relationships Reported in Medical Records.* Cell, 2018. **173**(7): p. 1692-1704.e11.
11. Lakhani, C.M., et al., *Repurposing large health insurance claims data to estimate genetic and environmental contributions in 560 phenotypes.* Nat Genet, 2019. **51**(2): p. 327-334.
12. Sudlow, C., et al., *UK biobank: an open access resource for identifying the causes of a wide range of complex diseases of middle and old age.* PLoS Med, 2015. **12**(3): p. e1001779.
13. Sudlow, C., et al., *UK biobank: an open access resource for identifying the causes of a wide range of complex diseases of middle and old age.* Plos med, 2015. **12**(3): p. e1001779.
14. Upadhyaya, S.G., et al., *Automated Diabetes Case Identification Using Electronic Health Record Data at a Tertiary Care Facility.* Mayo Clin Proc Innov Qual Outcomes, 2017. **1**(1): p. 100-110.
15. Lee, S.H., et al., *Estimating missing heritability for disease from genome-wide association studies.* Am J Hum Genet, 2011. **88**(3): p. 294-305.
16. Falconer, D.S., *The inheritance of liability to certain diseases, estimated from the incidence among relatives.* Annals of human genetics, 1965. **29**(1): p. 51-76.
17. Aitken, A.C., *Note on selection from a multivariate normal population.* Proceedings of the Edinburgh Mathematical Society, 1935. **4**(2): p. 106-110.
18. Reich, T., J.W. James, and C.A. Morris, *The use of multiple thresholds in determining the mode of transmission of semi-continuous traits.* Ann Hum Genet, 1972. **36**(2): p. 163-84.
19. Anderson, M.L., *As the Wind Blows: The Effects of Long-Term Exposure to Air Pollution on Mortality.* Journal of the European Economic Association, 2019.
20. Herrnstadt, E. and E. Muehlegger, *Air Pollution and Criminal Activity: Evidence from Chicago Microdata.* National Bureau of Economic Research Working Paper Series, 2015. **No. 21787**.
21. Schlenker, W. and W.R. Walker, *Airports, Air Pollution, and Contemporaneous Health.* The Review of Economic Studies, 2015. **83**(2): p. 768-809.
22. Deryugina, T., et al., *The Mortality and Medical Costs of Air Pollution: Evidence from Changes in Wind Direction.* Am Econ Rev, 2019. **109**(12): p. 4178-4219.

23. Zhang, Q., et al., *Transboundary health impacts of transported global air pollution and international trade.* Nature, 2017. **543**(7647): p. 705-709.
24. Baiocchi, M., J. Cheng, and D.S. Small, *Instrumental variable methods for causal inference.* Stat Med, 2014. **33**(13): p. 2297-340.
25. Paciorek, C.J., *The importance of scale for spatial-confounding bias and precision of spatial regression estimators.* Stat Sci, 2010. **25**(1): p. 107-125.
26. Hanks, E.M., et al., *Restricted spatial regression in practice: Geostatistical models, confounding, and robustness under model misspecification.* Environmetrics, 2015. **26**(4): p. 243-254.
