# Supplementary material for: Dissecting heritability, environmental risk, and air pollution causal effects using > 50 million individuals in MarketScan
Source: Nat Commun. 2024 Jun 25;15:5357. doi: 10.1038/s41467-024-49566-6 (PMC11199552; doi:10.1038/s41467-024-49566-6)
Supplement: Supplementary file 3 — Description of Additional Supplementary Files [file 41467_2024_49566_MOESM3_ESM.pdf]

Description of Additional Supplementary Files (The tables are available as Excel spreadsheet).

File name: Supplementary Data 1
Description: Accuracy of variance component estimates using SMILE and its sub-models. We simulated binary phenotypes for 50,000 or 250,000 nuclear families. We considered prevalence values of 0.001, 0.005, 0.01, 0.05, 0.1, and 0.4, and also continuous phenotype (denoted by Prevalence=1). We also varied variance component values $\sigma_g^2 = \{0, 0.3, 0.5\}$, $\sigma_s^2 = \{0, 0.01, 0.05\}$, $\sigma_{child}^2 = \{0, 0.05, 0.1, 0.2, 0.6\}$, and $\sigma_{par}^2 = \{0, 0.05, 0.2\}$, where σ_g^2 denotes genetic variance, σ_{par}^2 denotes shared parental environment, σ_{child}^2 denotes shared children's environment, and σ_s^2 denotes spatially correlated community-level environment. The parameter values are informed by the distribution of variance components estimated in the MarketScan dataset.

File name: Supplementary Data 2
Description: Variance components and causal effect estimates from 1083 diseases based on SMILE model. We report estimates and standard errors for the heritability (G), and the variance components of parent-shared (P), children-shared (C), and spatially-correlated community environment (S) from SMILE model. We also report odds ratio, their p-values, and 95% confidence interval for the causal effects of air pollution levels from the SMILE-2 model. All p-values are for two-sided hypothesis tests and are unadjusted for multiple comparisons.

File name: Supplementary Data 3
Description: Impact of noisy pedigrees and heritability estimates from SMILE model. We simulated families with adoptive and stepchildren, encoded them as biological children for both parents in the analyses, and assessed the impact on heritability estimates. Our results show the heritability estimates still remain unbiased and the confidence intervals of the estimates using noisy pedigrees still covers the true heritability values.

File name: Supplementary Data 4
Description: Variance components estimates and standard errors estimated using families enrolled for 10-12 years and families enrolled for 6-7 years in the MarketScan dataset. We assessed the impact of the length of enrollment on variance components estimates. We compared estimates using families enrolled for different lengths, and the results are highly concordant. Reassuringly, the length of enrollment has minimal impact on the variance components estimates.

File name: Supplementary Data 5
Description: Comparison of SMILE model variance components estimates using ICD9/10 codes only to using both diagnosis and prescription codes. The resulting estimates of the heritability (G), and variance components of parental-shared (P), children-shared (C), and spatially-correlated environment (S) are very similar using either phenotype definitions. Importantly, adding spatial random effects to the model still corrected for the upward bias due to correlated community-level environment and our main conclusions remain regardless of the phenotype definitions used.

File name: Supplementary Data 6
Description: Drug codes that are used to define type 2 diabetes (T2D). We consider defining the T2D phenotype using both the drug codes and diagnosis codes and use the newly defined phenotypes to estimate T2D heritability, and the variance components of parental-shared, children-shared, and spatially-correlated community-level environment. The results remain very similar.

File name: Supplementary Data 7
Description: Environmental exposures at county and MSA level for MarketScan nuclear families. Raw values for the covariates are shown. In the analysis, the covariates were centered and scaled for all fitted models.

File name: Supplementary Data 8
Description: Demographic variables extracted from the ACS 5-year survey. The demographic fields were extracted from the 2015 ACS 5-year suvey using the 'tidycensus' R package. The following R command loads ACS variables into a dataframe with 3 columns (Name, Label, Concept): "*library(tidycensus); acsv <-*

load_variables(2015, ""acs5/subject"",cache=TRUE)". The field names, labels, and concepts corresponding to the census data used in our study, as outputted by the R command. The last column (Notes), is the author's laymen description of the field groups.
