# Supplementary material for: Dissecting heritability, environmental risk, and air pollution causal effects using > 50 million individuals in MarketScan
Source: Nat Commun. 2024 Jun 25;15:5357. doi: 10.1038/s41467-024-49566-6 (PMC11199552; doi:10.1038/s41467-024-49566-6)
Supplement: Supplementary file 5 — Reporting Summary [file 41467_2024_49566_MOESM5_ESM.pdf]

nature portfolio

Corresponding author(s): Bibo Jiang, Dajiang J. Liu

Last updated by author(s): 05/19/2024

Reporting Summary

Nature Portfolio wishes to improve the reproducibility of the work that we publish. This form provides structure for consistency and transparency in reporting. For further information on Nature Portfolio policies, see our Editorial Policies and the Editorial Policy Checklist.

Statistics

For all statistical analyses, confirm that the following items are present in the figure legend, table legend, main text, or Methods section.

n/a	Confirmed	
☐	☒	The exact sample size (*n*) for each experimental group/condition, given as a discrete number and unit of measurement
☐	☒	A statement on whether measurements were taken from distinct samples or whether the same sample was measured repeatedly
☐	☒	The statistical test(s) used AND whether they are one- or two-sided<br>*Only common tests should be described solely by name; describe more complex techniques in the Methods section.*
☐	☒	A description of all covariates tested
☐	☒	A description of any assumptions or corrections, such as tests of normality and adjustment for multiple comparisons
☐	☒	A full description of the statistical parameters including central tendency (e.g. means) or other basic estimates (e.g. regression coefficient) AND variation (e.g. standard deviation) or associated estimates of uncertainty (e.g. confidence intervals)
☐	☒	For null hypothesis testing, the test statistic (e.g. *F*, *t*, *r*) with confidence intervals, effect sizes, degrees of freedom and *P* value noted<br>*Give P values as exact values whenever suitable.*
☒	☐	For Bayesian analysis, information on the choice of priors and Markov chain Monte Carlo settings
☒	☐	For hierarchical and complex designs, identification of the appropriate level for tests and full reporting of outcomes
☐	☒	Estimates of effect sizes (e.g. Cohen's *d*, Pearson's *r*), indicating how they were calculated

Our web collection on statistics for biologists contains articles on many of the points above.

Software and code

Policy information about availability of computer code

Data collection	We did not collect any new data for this study. The analysis was based on existing data from the MarketScan dataset.

Data analysis	The software package implementing the SMILE and SMILE-2 model is available at https://github.com/dan11mcguire/smile<br>R Project for Statistical Computing version 4.0 https://www.r-project.org;<br>R packages:<br>tidyr R package version 1.1.2<br>dplyr R package version 1.0.2<br>tidyverse R package version 1.3.0<br>ggpubr R package version 0.4.0<br>reshape2 R package version 1.4.4<br>ggplot2 R package version 3.3.2<br>RColorBrewer R package version 1.1-2<br>data.table R package version 1.13.0<br>patchwork R package version 1.1.0.9000<br>tidycensus R package version 1.6.2<br>sf R package version 1.0.3<br>TMB R package version 1.9.11<br>Matrix R package version 1.6-1.1<br>Rcpp R package version 1.0.12<br>spdep R package version 1.3-3

For manuscripts utilizing custom algorithms or software that are central to the research but not yet described in published literature, software must be made available to editors and reviewers. We strongly encourage code deposition in a community repository (e.g. GitHub). See the Nature Portfolio guidelines for submitting code & software for further information.

Data

Policy information about availability of data

All manuscripts must include a data availability statement. This statement should provide the following information, where applicable:

- Accession codes, unique identifiers, or web links for publicly available datasets
- A description of any restrictions on data availability
- For clinical datasets or third party data, please ensure that the statement adheres to our policy

The raw data from Truven MarketScan are available for licensed users. A user license could be obtained by following the instructions at https://marketscan.truvenhealth.com/marketscanportal/.

We provide all the data that support the findings of this study in this published article (and its supplementary information files). All the variance components estimated from SMILE for 1,083 binary diseases as defined by the PheWAS code are available in Supplementary Data 2. All the causal effects of PM2.5 and NO2 air pollution for 1,083 PheWAS codes are also available in Supplementary Data 2. Variance components estimates and standard errors estimated using families enrolled for 10-12 years and families enrolled for 6-7 years in the MarketScan dataset can be found in Supplementary Data 4. Drug codes that are used to define type 2 diabetes (T2D) can be found in Supplementary Table 6. Environmental exposures at county and MSA level for MarketScan nuclear families can be found in Supplementary Data 7. Demographic variables extracted from the ACS 5-year survey are provided in Supplementary Data 8.

Research involving human participants, their data, or biological material

Policy information about studies with human participants or human data. See also policy information about sex, gender (identity/presentation), and sexual orientation and race, ethnicity and racism.

Reporting on sex and gender	We included the participant reported sex as one of the individual-level fixed-effect covariates.
Reporting on race, ethnicity, or other socially relevant groupings	We include racial distributions at the county or MSA level from the 2015 ACS community survey as one of the community level risk factors to calculate the community-level environmental contribution for each disease at each MarketScan location.
Population characteristics	We include averaged minimum and maximum monthly temperature and precipitation levels, averaged PM2.5 and NO2 air pollution, as well as sociodemographic variables for median income, population density, poverty rates, and education levels at the county or MSA level from the 2015 ACS community survey as other community level risk factors to calculate the total community-level environmental contribution for each disease at each MarketScan location.<br><br>We also (1) limited our analysis to families enrolled in the database for at least 6 years, (2) to families where all children are at least 10 years old at the time of entry into the database, and (3) excluded families where the age at enrollment of the youngest family member was less than the 5th percentile of the age of diagnosis for the phenotype of interest and (4) included age and age2 as covariates in both SMILE and SMILE-2 to account for the impact of age.
Recruitment	There was no recruitment of participants for this study.
Ethics oversight	This study is deemed non-human subject research and approved by Penn State College of Medicine IRB.

Note that full information on the approval of the study protocol must also be provided in the manuscript.

Field-specific reporting

Please select the one below that is the best fit for your research. If you are not sure, read the appropriate sections before making your selection.

☒ Life sciences ☐ Behavioural & social sciences ☐ Ecological, evolutionary & environmental sciences

For a reference copy of the document with all sections, see nature.com/documents/nr-reporting-summary-flat.pdf

Life sciences study design

All studies must disclose on these points even when the disclosure is negative.

Sample size

We inferred family structure from the relationships of each enrollee to the designated policy holders in each family, and performed stringent data quality checks to ensure the accuracy of the inferred familial relationships. Specifically, we assume that "Employee" and "Spouse" are the biological parents of the enrollees encoded as "Child/Other" within the family. We selected for analysis nuclear families which were enrolled for at least 6 years within the database. We also required that the youngest sibling in each selected family be at least 10 years old at the beginning of their 6+ year enrollment period. To maximize the validity of the assumed biological relationships within a family, we removed families where the "Employee" and "Spouse" were of the same sex, and families where either "Employee" or "Spouse" were not older than the "Children" by at least 14 years. We also removed families where the designated "Employee" had multiple "Spouses" with different enrollment ID's, as in this situation it is unclear which Spouse (if any) may be the biological parent of the children. This curated dataset consisted of 257,620 families for which we carried forward the downstream analyses.

Data exclusions

To maximize the validity of the assumed biological relationships within a family, we removed families where the "Employee" and "Spouse" were of the same sex, and families where either "Employee" or "Spouse" were not older than the "Children" by at least 14 years. We also removed families where the designated "Employee" had multiple "Spouses" with different enrollment ID's, as in this situation it is unclear which Spouse (if any) may be the biological parent of the children.

Replication

We compared and replicated our results with several published heritability studies, including
1. An independent study using MarketScan database MS1, but analyzed without modeling the shared community-level environment;
2. A study that repurposed EHR data from New York State NY;
3. A study CaTCH that analyzed twins from EHR data to estimate genetic and environmental contributions, and
4. Heritability estimates based upon GWAS summary statistics from UK Biobank (LDSC-UKB).

Randomization

No randomization was conducted, since randomization is not applicable for association studies in case control and population-based biobanks.

Blinding

No blinding was conducted, since blinding is not applicable for association studies in case control and population-based biobanks. No intervention is implemented.

Reporting for specific materials, systems and methods

We require information from authors about some types of materials, experimental systems and methods used in many studies. Here, indicate whether each material, system or method listed is relevant to your study. If you are not sure if a list item applies to your research, read the appropriate section before selecting a response.

Materials & experimental systems

n/a	Involved in the study
☒	☐ Antibodies
☒	☐ Eukaryotic cell lines
☒	☐ Palaeontology and archaeology
☒	☐ Animals and other organisms
☒	☐ Clinical data
☒	☐ Dual use research of concern
☒	☐ Plants

Methods

n/a	Involved in the study
☒	☐ ChIP-seq
☒	☐ Flow cytometry
☒	☐ MRI-based neuroimaging

Plants

Seed stocks	*Report on the source of all seed stocks or other plant material used. If applicable, state the seed stock centre and catalogue number. If plant specimens were collected from the field, describe the collection location, date and sampling procedures.*
Novel plant genotypes	*Describe the methods by which all novel plant genotypes were produced. This includes those generated by transgenic approaches, gene editing, chemical/radiation-based mutagenesis and hybridization. For transgenic lines, describe the transformation method, the number of independent lines analyzed and the generation upon which experiments were performed. For gene-edited lines, describe the editor used, the endogenous sequence targeted for editing, the targeting guide RNA sequence (if applicable) and how the editor was applied.*
Authentication	*Describe any authentication procedures for each seed stock used or novel genotype generated. Describe any experiments used to assess the effect of a mutation and, where applicable, how potential secondary effects (e.g. second site T-DNA insertions, mosiacism, off-target gene editing) were examined.*
